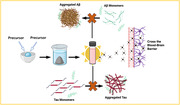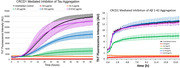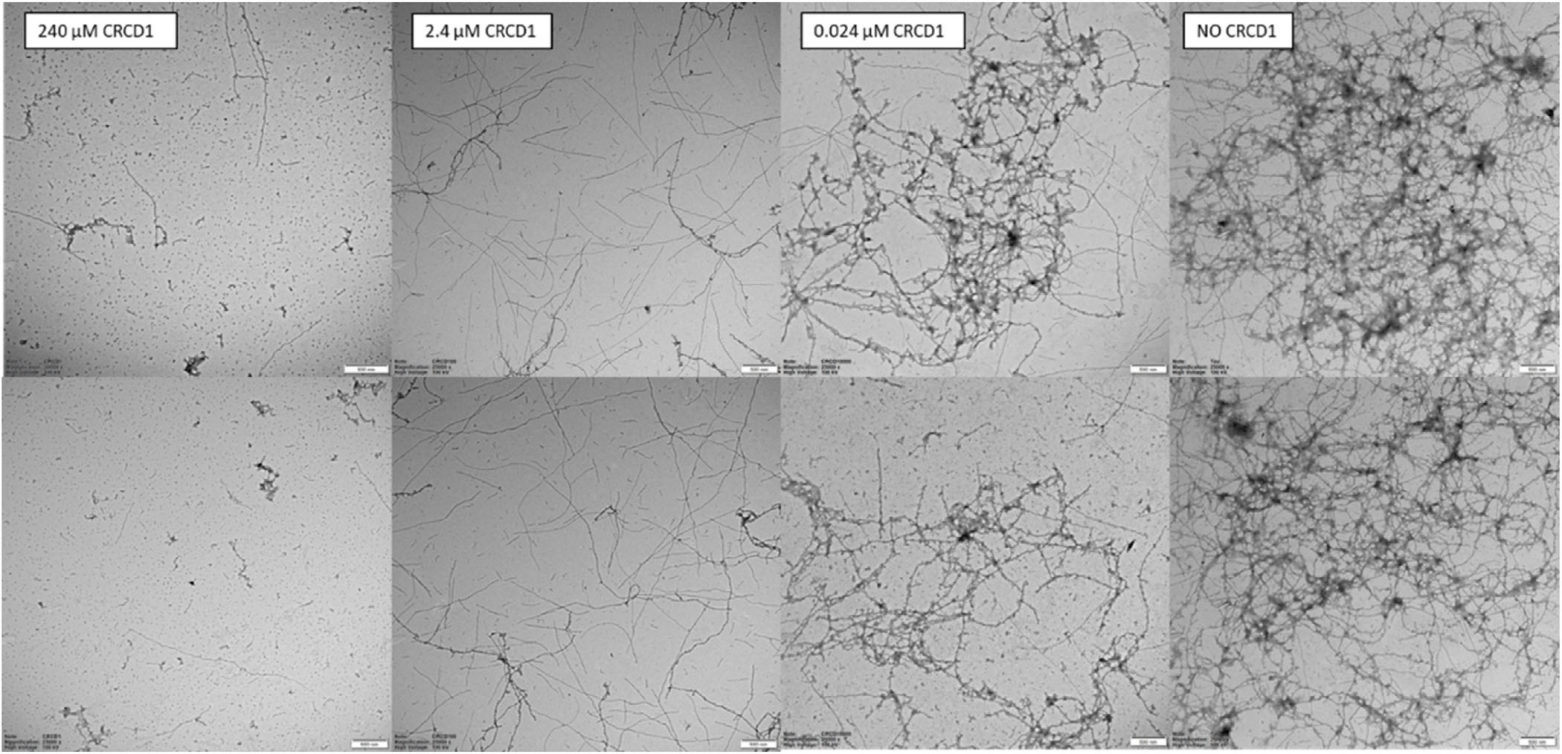# Development of Diverse Carbon Dots to Inhibit Protein Aggregation in Alzheimer’s Disease

**DOI:** 10.1002/alz.087705

**Published:** 2025-01-09

**Authors:** Nathan Scott Smith, Hannah J Burr, Wei Zhang, Chunyu Wang

**Affiliations:** ^1^ Rensselaer Polytechnic Institute, Troy, NY USA; ^2^ University of Miami, Miami, FL USA

## Abstract

**Background:**

Alzheimer’s Disease (AD) is the leading form of senile dementia, affecting ∼6 million Americans and having a national economic impact of $321 billion, numbers expected to double by 2050. The major pathological hallmarks of AD include Amyloid Beta (Aβ) plaques and Tau neurofibrillary tangles (NFT). The first goal of this research was to develop novel forms of carbon dots (CD) using various precursors. The next goal was to use these unique CDs to inhibit the aggregated hallmarks of AD. Some dots were also conjugated to drug compounds such as chalcones and memantines.

**Method:**

We synthesized CDs from precursors such as urea, Congo red, memantine, and citric acid, according to the Muffle furnace method. We then aimed to utilize the newly synthesized CDs to inhibit tau and Aβ aggregation *in vitro*. Thioflavin T (ThT) aggregation assays were then selected to quantify the protein aggregation and was verified via atomic force microscopy (AFM) and transmission electron microscopy (TEM) for visualization. Zebrafish models were chosen to confirm blood brain barrier (BBB) penetration.

**Result:**

Almost all CDs demonstrated inhibition of both tau and Aβ aggregation, suggesting their use as dual inhibitors. Some CDs demonstrated IC_50_ values for inhibition, as low as 0.2 ± 0.1 mg/mL for tau and 2 ± 2 mg/mL for Aβ aggregation. Many of these aggregates were verified using AFM technology and TEM technology to acquire photos of aggregates. As the concentration of CDs increased, the number of visible protein aggregates decreased. Some of the CDs synthesized also demonstrated the ability to penetrate the BBB as shown during experimentation with zebrafish.

**Conclusion:**

Overall, the data suggests CDs have a unique ability to inhibit the aggregation of both AD hallmarks. This data encourages investing further research into CDs for the future treatment of AD.